# Heteronuclear and homonuclear radio-frequency-driven recoupling

**DOI:** 10.5194/mr-2-343-2021

**Published:** 2021-05-28

**Authors:** Evgeny Nimerovsky, Kai Xue, Kumar Tekwani Movellan, Loren B. Andreas

**Affiliations:** Department of NMR-based Structural Biology, Max Planck Institute for Biophysical Chemistry, Am Fassberg 11, Göttingen, Germany

## Abstract

The radio-frequency-driven recoupling (RFDR) pulse sequence is used in magic-angle spinning (MAS) NMR to recouple homonuclear dipolar interactions. Here we show simultaneous recoupling of both the heteronuclear and homonuclear dipolar interactions by applying RFDR pulses on two channels. We demonstrate the method, called HETeronuclear RFDR (HET-RFDR), on microcrystalline SH3 samples at 10 and 55.555 kHz MAS. Numerical simulations of both HET-RFDR and standard RFDR sequences allow for better understanding of the influence of offsets and paths of magnetization transfers for both HET-RFDR and RFDR experiments, as well as the crucial role of XY phase cycling.

## 1 Introduction

Magic-angle spinning (MAS) NMR spectroscopy is used to obtain atomic resolution spectra of materials and biological molecules in the solid state, by removal of the broadening associated with anisotropic dipolar couplings and other interactions. Under control of radio frequency pulses, dipolar interactions can be switched on, or recoupled, in order to correlate nearby spins or to accurately determine internuclear distances. Recoupling sequences can be broadly categorized as homonuclear (Meier and Earl, 1986; Tycko and Dabbagh, 1990; Gullion and Vega, 1992; Bennett et al., 1992; Ok et al., 1992; Zhang et al., 2020; Gelenter et al., 2020; Takegoshi et al., 2001; Szeverenyi et al., 1982; Hou et al., 2011b, 2013; Carravetta et al., 2000; Bennett et al., 1998; Nielsen et al., 2011) or heteronuclear (Gelenter et al., 2020; Gullion and Schaefer, 1989; Jaroniec et al., 2002; Hing et al., 1992; Hartmann and Hahn, 1962; Rovnyak, 2008; Metz et al., 1994; Hediger et al., 1994; Hou et al., 2011a; Brinkmann and Levitt, 2001; Gelenter and Hong, 2018; Zhang et al., 2016; Nielsen et al., 2011).

The recoupling of the homonuclear dipolar interactions with a train of π pulses every rotor period was originally introduced by Gullion and Vega (1992) and Bennett et al. (1992). Since then, the homonuclear radio-frequency-driven recoupling (RFDR) sequence (Bennett et al., 1992) has been successfully applied for the qualitative and quantitative determinations of the dipolar spin correlations in materials (Saalwächter, 2013; Messinger et al., 2015; Fritz et al., 2019; Roos et al., 2018; Nishiyama et al., 2014a; Wong et al., 2020; Hellwagner et al., 2018; Pandey and Nishiyama, 2018) and biomolecular samples (Zheng et al., 2007; Tang et al., 2011; Shen et al., 2012; Pandey et al., 2014; Grohe et al., 2019; Andreas et al., 2015; Petkova et al., 2002; Aucoin et al., 2009; Zinke et al., 2018; Zhang et al., 2017; Zhou et al., 2012; Jain et al., 2017; Colvin et al., 2015; Shi et al., 2015; Daskalov et al., 2021). Sun et al. (1995) showed that the RFDR pulse sequence element could also be used as a part of the SPICP experiment (Wu and Zilm, 1993) for removing the undesired effect of the chemical shift terms to zero order.

Depending on the assumptions (Bennett et al., 1992; Gullion and Vega, 1992; Ishii, 2001), two different average Hamiltonian theory (AHT; Haeberlen and Waugh, 1968; Maricq, 1982) descriptions have been detailed for RFDR. In both, homonuclear dipolar recoupling occurs via a rotor-synchronized train of π pulses, with one pulse each rotor period (Bennett et al., 1992) on a single channel. In the first case, delta π pulses are assumed (Bennett et al., 1992). The efficiency of recoupling is linked with the rotational resonance conditions (Bennett et al., 1992, 1998) and depends on the ratio between the chemical shift offset difference and the MAS rate. In the second theoretical description, the effects of finite π pulses are considered (Bennett et al., 1992; Ishii, 2001; Nishiyama et al., 2014b; Zhang et al., 2015; Brinkmann et al., 2002; Ji et al., 2020). The efficiency of recoupling in this case depends on a duty factor (Ishii, 2001), defined as the fraction of the rotor period occupied by the π pulse. The RFDR pulses are applied according to a variety of XY phase cycling schemes, which have been analyzed with the intent to suppress imperfections associated with offset differences, radio frequency (rf)-field inhomogeneity, and second-order average Hamiltonian terms between different anisotropic interactions (Zhang et al., 2015).

The full high-field truncated dipolar Hamiltonian of the homonuclear $I_{2}$ spin system is represented as follows:

1 $$H_{D,\mathrm{Full}}^{\mathrm{II}}=\omega_{D,12}(t)\left[3I_{z1}I_{z2}-{\bar{I}}_{1}\cdot {\bar{I}}_{2}\right],$$

where $\omega_{D,12}(t)$ is a periodic time-dependent function (Olejniczak et al., 1984) that depends on the positions of spins $I_{1}$ and $I_{2}$ within the rotor. This Hamiltonian is subsequently referred to as the full Hamiltonian and contains only the A and B terms of the dipolar alphabet (Slichter, 1990).

An interesting conclusion can be obtained if we simplify Eq. (1). The dipolar Hamiltonian during RFDR can be simplified (in the absence of other interactions) by considering that ${\bar{I}}_{1}\cdot {\bar{I}}_{2}$ commutes with the secular part ( $I_{z1}I_{z2}$ ) and with the rf-field Hamiltonian. At the end of each rotor period, the oscillatory $\omega_{D,12}(t)$ term ensures zero total evolution. The simplified Eq. (1) is

2 $$H_{D,M}^{\mathrm{II}}=1.5\omega_{D,12}(t)2I_{z1}I_{z2}.$$

Comparing Eq. (2) with the full dipolar Hamiltonian of the heteronuclear *IS* spin system (Mehring, 1983),

3 $$H_{D,\mathrm{Full}}^{IS}=\omega_{D,12}(t)2I_{z}S_{z},$$

we notice that the difference between Eq. (3) and Eq. (1) is a factor of 1.5. Note that we have made the substitution of $I_{z1}$ to $I_{z}$ and $I_{z2}$ to $S_{z}$ , while the dipolar function, $\omega_{D,12}(t)$ , has been kept the same. Such comparison suggests a HETeronuclear RFDR (HET-RFDR), which should have a scaling of 1.5 as compared with the homonuclear case.

**Figure 1:**
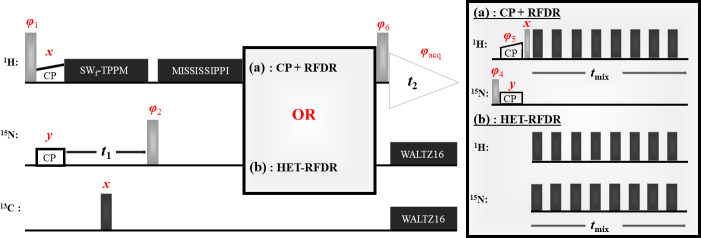
Two versions of the (H)N(H)H pulse sequence are shown. The first, **(a)**, is the standard implementation with CP + RFDR. The second, **(b)**, instead uses the new HET-RFDR recoupling element. Light grey pulses represent π /2 pulses, whereas dark grey pulses represent π pulses. The ramped CP transfer from proton to nitrogen as well as from nitrogen to proton in **(a)** is indicated with constant power on the nitrogen channel and a ramp in power on the proton channel. During the indirect dimension ( $t_{1}$ ), SW ${}_{f}$ –TPPM decoupling is applied at 55 kHz, respectively. A single π pulse in the middle of $t_{1}$ decouples carbon. Water suppression is implemented with the MISSISSIPPI (Zhou and Rienstra, 2008) sequence. During acquisition, WALTZ16 (Thakur et al., 2006) decoupling is applied on nitrogen and carbon channels. The phases are $\varphi_{1}=x,-x$ ; $\varphi_{\mathrm{acq}}=y,-y,-y,y,-y,y,y,-y$ . In **(a)** the phases are $\varphi_{2}=x$ ; $\varphi_{4}=x,x,-x,-x$ ; $\varphi_{5}=y,y,y,y,-y,-y,-y,-y$ ; $\varphi_{6}=x$ . In **(b)** the phases are $\varphi_{2}=x,x,-x,-x$ ; $\varphi_{6}=x,x,x,x,-x,-x,-x,-x$ . RFDR π pulses on both channels use the XY8 scheme (Gullion et al., 1990).

In this article we investigate spin dynamics under HET-RFDR, in which RFDR π pulses are applied simultaneously on two channels (Fig. 1). We demonstrate simultaneous heteronuclear and homonuclear transfers using HET-RFDR applied to α -PET-labeled SH3 (Movellan et al., 2019) at 10 and 55.555 kHz MAS.

We perform and compare a numerical operator analysis of both RFDR and HET-RFDR experiments under different simulated conditions. This numerical analysis allows us to define the conditions under which homonuclear and heteronuclear RFDR polarization transfers have similar behaviors, to understand the paths through which the signals are transferred between operators, and to understand the crucial role of 90 ${}^{\circ }$ phase alternation (XY-4, XY-8, etc.) (Ishii, 2001; Nishiyama et al., 2014b; Zhang et al., 2015; Hellwagner et al., 2018) for both RFDR and HET-RFDR recoupling.

## 2 HET-RFDR experiments

Figure 1 shows two 2D (H)N(H)H pulse sequences used to evaluate the HET-RFDR transfer. For both sequences, the transfer from proton to nitrogen is implemented with ramped cross polarization (CP), and then the nitrogen dimension is encoded ( $t_{1}$ ) for 2D spectra. In Fig. 1a, the transfer to structurally interesting protons is implemented with N to H CP followed by H–H RFDR. In Fig. 1b, the same transfer is implemented with a single HET-RFDR period. The HET-RFDR transfer avoids the back CP step. Instead, nitrogen polarization is placed along the $\hat{z}$ axis and transferred to directly bonded proton spins and at the same time to remote proton spins with the simultaneous application of the π pulses on the proton and nitrogen channels.

**Figure 2:**
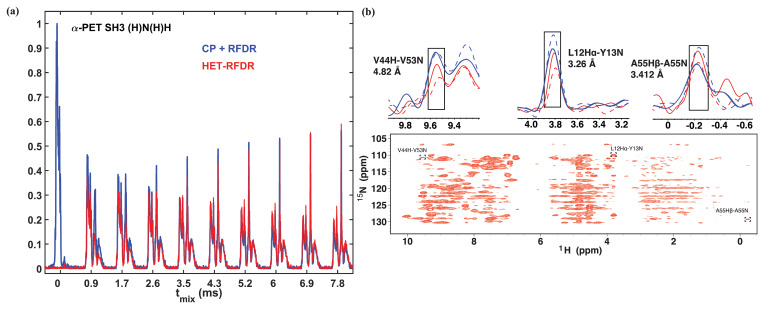
1D **(a)** and 2D **(b)** (H)N(H)H spectra of α -PET-labeled SH3. For all spectra, the first CP from proton to nitrogen was performed with 1.05 ms. **(a)** 1D spectra with different sequences used for the second transfer: CP + RFDR (blue) and HET-RFDR (red). For CP + RFDR, 0.55 ms of CP was used. For both RFDR and HET-RFDR, $t_{\mathrm{mix}}$ of 0, 0.846, 1.728, 2.592, 3.456, 4.32, 5.184, 6.048, 6.912, 7.7776 ms are shown. **(b)** 2D HET-RFDR at 3.456 ms of mixing time. Spectra were recorded with a 600 MHz Bruker instrument equipped with a 1.3 mm probe and an MAS frequency of 55 kHz. The widths of π pulses on proton and nitrogen channels were 5.8 and 6.6 µs , respectively. The 1D slices show the intensities of three selected peaks. CP + RFDR (blue) and HET-RFDR (red) at 1.154 ms (dashed lines) and 3.456 ms (solid lines) mixing are displayed. The experimental parameters are detailed in Tables 1 and 2 in the Experimental methods section. XY8 phase cycling was used.

Figure 2 compares the 1D and 2D spectra obtained with the two sequences of Fig. 1. In Fig. 2a, the 1D signal is shown as a function of RFDR mixing time. For the standard sequence (blue), the N to H CP was 0.55 ms. The HET-RFDR signal is shown in (red). Without RFDR mixing, the CP + RFDR detects directly bonded amide protons (Fig. 2a, red with zero mixing time), and zero signal occurs for HET-RFDR (Fig. 2a, blue with zero mixing time) since the signal is on nitrogen. With increasing RFDR mixing, the signal is transferred from directly bonded amide protons to remote protons for the CP + RFDR sequence (red), whereas simultaneous transfer from nitrogen spins to amide protons and from amide protons to remote protons occurs with HET-RFDR (blue). For the directly bonded amide protons, the HET-RFDR polarization transfer achieves only ∼40 % of the CP signal. This occurs at 0.846 ms mixing (second red spectrum). However, with increased mixing of about 3 ms, HET-RFDR reaches the same efficiency as the standard sequence. This is notable since transfer over long distances has been implemented with ∼3 ms mixing for deuterated samples (Grohe et al., 2019; Linser et al., 2014).

Structurally interesting cross-peaks are indeed observed in the 2D HET-RFDR spectrum shown in Fig. 2b at 3.456 ms mixing. For example, we have observed the amide–amide contact between V44 and V53, which is 4.82 Å in the crystal (pdb code 2NUZ; Castellani et al., 2002). The amide to side chain contact of a A55 N to Hβ (3.41 Å) is also indicated in the figure, along with a sequential contact from Y13 ${}^{15}N$ to L12 ${}^{1}H\alpha$ , which is 3.26 Å. These peaks are boxed in Fig. 2b, and the 1D slices are shown above the 2D spectra. For comparison, in 1D slices we show CP + RFDR (blue) and HET-RFDR (red) intensities of these three peaks for two different mixing times: 1.154 ms (dashed) and 3.456 ms (solid). Both methods provide similar intensities at long mixing time, whereas at shorter mixing times, CP + RFDR provides higher intensities for short-range distances.

**Figure 3:**
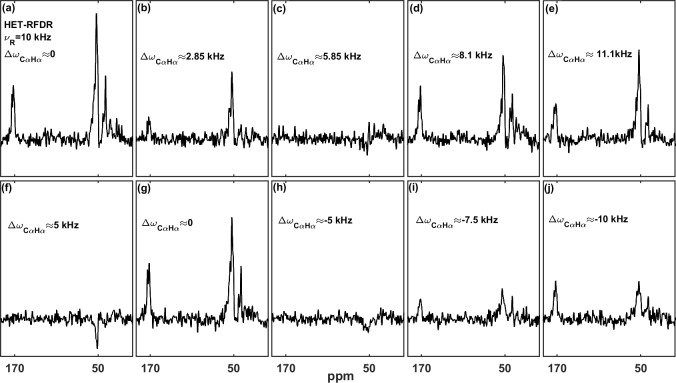
The influence of the carbon and proton offsets on proton–carbon HET-RFDR polarization transfers at 4.8 ms mixing. α -PET-labeled SH3 was used with 10 kHz MAS with a 600 MHz spectrometer using a 1.3 mm probe. The widths of π pulses on proton and carbon channels were 5.8 us and 6.6 us, respectively. For **(a–e)** the proton carrier frequency was set to 4.6 ppm, and carbon carrier frequency was set to 51 ppm **(a)**, 70 ppm **(b)**, 90 ppm **(c)**, 105 ppm **(d)**, and 125 ppm **(e)**. For **(f–j)**, the carbon carrier frequency was set to 83.66 ppm, and the proton carrier frequency was set to 4.6 ppm **(f)**, 12.933 ppm **(g)**, 21.26 ppm **(h)**, 25.43 ppm **(i)**, and 29.6 ppm **(j)**. The indicated offset differences, $\Delta \Omega_{C\alpha H\alpha }=\Omega_{C\alpha }-\Omega_{H\alpha }$ , in kilohertz (kHz), were calculated based on typical isotropic chemical shifts of C ${}_{\alpha }$ (51 ppm) and H ${}_{\alpha }$ (4.6 ppm) with a 600 MHz spectrometer. The experimental parameters are detailed in Tables 1 and 2 in the Experimental methods section. The 1D HET-RFDR sequence is shown in the Supplement (Fig. S1). XY8 phase cycling was used.

At 55.555 kHz MAS on a 600 MHz instrument, the chemical shift offsets can always be much smaller than the spinning frequency. At a lower MAS frequency, the offsets become important for HET-RFDR. The recoupling then depends on a heteronuclear “offset difference” that we define as $\Delta \Omega_{ij}=\Omega_{i}-\Omega_{j}$ , where $\Omega_{i}$ and $\Omega_{j}$ are the offsets on each channel (the difference between the Larmor frequency of the spin and the carrier frequency; Bak et al., 2000). When $\Omega_{i}=\Omega_{j}=0$ or when $\Delta \Omega_{ij}=\Omega_{i}-\Omega_{j}\approx n\nu_{R}$ ( n=0 , ±1 , ±2… ), the HET-RFDR polarization transfer reaches local maximal intensities. However, when $\Delta \Omega_{ij}=\Omega_{i}-\Omega_{j}\approx 0.5n\nu_{R}$ ( n=±1 , ±3… ), the HET-RFDR polarization transfer reaches local minima. The experimental confirmation of this is shown in Fig. 3, where the effect of different proton and carbon offsets is explored for proton–carbon HET-RFDR spectra. The spinning frequency was reduced to 10 kHz MAS for these measurements and the signal detected on the carbon channel. The 1D HC HET-RFDR pulse sequence is shown in the Supplement (Fig. S1).

Figure 3a–e depict the HET-RFDR spectra when the carbon carrier frequency is changed (numbers show the offset from the α carbon at ∼53 ppm), whereas the α proton offset is kept at 0 kHz (at 4.6 ppm). While heteronuclear transfer is detected at zero offset (Fig. 3a) or with 11.1 kHz carbon offset (Fig. 3e), the signal remains in the noise when the carbon offset is 5.85 kHz (Fig. 3c).

A similar effect can be detected when the proton carrier frequency is changed (increased from 4.6 ppm), but this time the carbon offset is set to 5 kHz from C α (83.66 ppm) to show that it is the offsets on both channels ( $\Delta \Omega_{C\alpha H\alpha }$ ) that are important (Fig. 3f–j). The series of spectra show local minimal transfers at offset differences of 5 kHz (Fig. 3f) and -5 kHz (Fig. 3h) and local maximal polarization transfers at differences of 0 (Fig. 3g) and -10 kHz (Fig. 3j).

## 3 Numerical operator analysis

To comprehend the mechanism underlying the transfers during the HET-RFDR and also the well-known RFDR pulse sequence, we use a numerical simulation approach. We identify the conditions under which the heteronuclear and homonuclear spin systems under HET-RFDR and RFDR sequences have similar behaviors. Considering the evolution of the different spin systems through HET-RFDR and RFDR during the first two rotor periods, we identify the operators that are involved in the polarization transfer.

**Figure 4:**
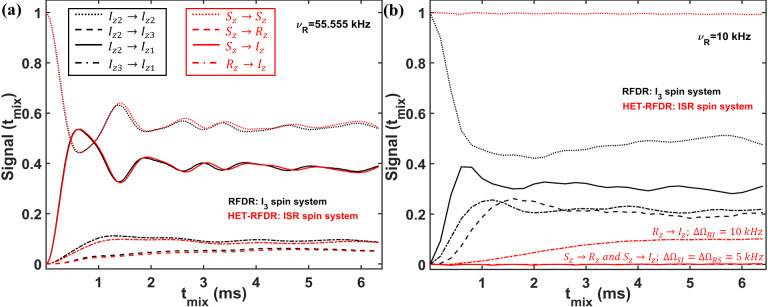
Comparison of the simulated RFDR and HET-RFDR signals. $I_{3}$ (three homonuclear spins, black lines) and *ISR* (three different spin types, red lines) for 55.555 kHz **(a)** and 10 kHz **(b)** MAS. An rf field of 83 kHz is used (6 µs of the widths of π pulses). The vertical axis shows the intensities of the starting and transferred signals between different operators of $I_{3}$ and *ISR* spin systems, respectively (the initial operator → the measured operator): $I_{z2}\to I_{z2}$ and $S_{z}\to S_{z}$ – (dotted lines); $I_{z2}\to I_{z3}$ and $S_{z}\to R_{z}$ – (dashed lines); $I_{z2}\to I_{z1}$ and $S_{z}\to I_{z}$ – (solid lines); $I_{z3}\to I_{z1}$ and $R_{z}\to I_{z}$ – (dashed–dotted lines). For both spin systems, the offset ( Ω ) and CSA (chemical shift anisotropy) values are [-3;2;7] (kHz) and [5.2;2.5;3] . The dipolar coupling constants for the homonuclear spin system ( $I_{3}$ ) are $\nu_{12,D}=7.333$ kHz, $\nu_{13,D}=2$ kHz, $\nu_{23,D}=0.333$ kHz. For the *ISR* spin system, all dipolar constants are 1.5 times larger: $\nu_{IS,D}=11$ kHz, $\nu_{IR,D}=3$ kHz, $\nu_{SR,D}=0.5$ kHz. The simulated measurements occur every two rotor periods. XY8 phase cycling was used. $I_{z1}\to I_{z1}$ , $I_{z3}\to I_{z3}$ , $I_{z}\to I_{z}$ and $R_{z}\to R_{z}$ are not shown.

To identify the conditions under which the HET-RFDR and RFDR sequences have similar and different behaviors, we simulated a three-spin system at high (55.555 kHz) and low (10 kHz) MAS frequencies. In Fig. 4, we compare the RFDR transferred signals for $I_{3}$ (a homonuclear three-spin system, black lines) and HET-RFDR transferred signals for *ISR* (three different types of spins with the names I , S , and R ; red lines) spin systems. At 55.555 kHz MAS when the offset difference is small compared to the MAS rate, the behavior of the homonuclear $I_{3}$ spin system is similar to the behavior of the heteronuclear *ISR* spin system (Fig. 4a). However, when the MAS rate is low (10 kHz) and the offset difference cannot be neglected, the behaviors of these spin systems are completely different (Fig. 4b). For the homonuclear spin system ( $I_{3}$ ), the polarization transfers are efficient for all dipolar pairs (black lines), whereas for the heteronuclear spin system (*ISR*) the HET-RFDR polarization transfer is detected between R and I spins (Fig. 4b, dashed–dotted red line) only. For this *RI* pair, the offset difference was chosen as 10 kHz, whereas for the other spin pairs (*SI*, *RS*), the offset differences were set to 5 kHz. These simulations show a special condition of $∼0.5\nu_{R}$ of offset difference for the heteronuclear spins under which the transfer obtains local/global minima values. The simulations are in full agreement with the experiments, which are shown in Fig. 3. Another interesting observation can be made from the influence of the offset difference on the RFDR transfer for the homonuclear $I_{3}$ spin system (Fig. 4b, black lines). For 5 kHz of offset difference, the RFDR polarization transfer between $I_{z2}$ and $I_{z3}$ spins is significantly faster with 10 kHz MAS (Fig. 4b, dashed black line) than at 55.555 kHz MAS (Fig. 4a, dashed black line). Since the duty factor is decreased with decreasing MAS frequency (Ishii, 2001), i.e., 0.33 for 55.555 kHz MAS and 0.06 for 10 kHz MAS, the opposite behavior is expected if one considers only the effect of finite pulses in the RFDR experiment (Ishii, 2001). It indicates that when the offset difference cannot be neglected with respect to the MAS rate, it has a significant influence on the RFDR transfer efficiency between homonuclear spins despite the significant remoteness from the rotational resonance condition (Bennett et al., 1992, 1998).

**Figure 5:**
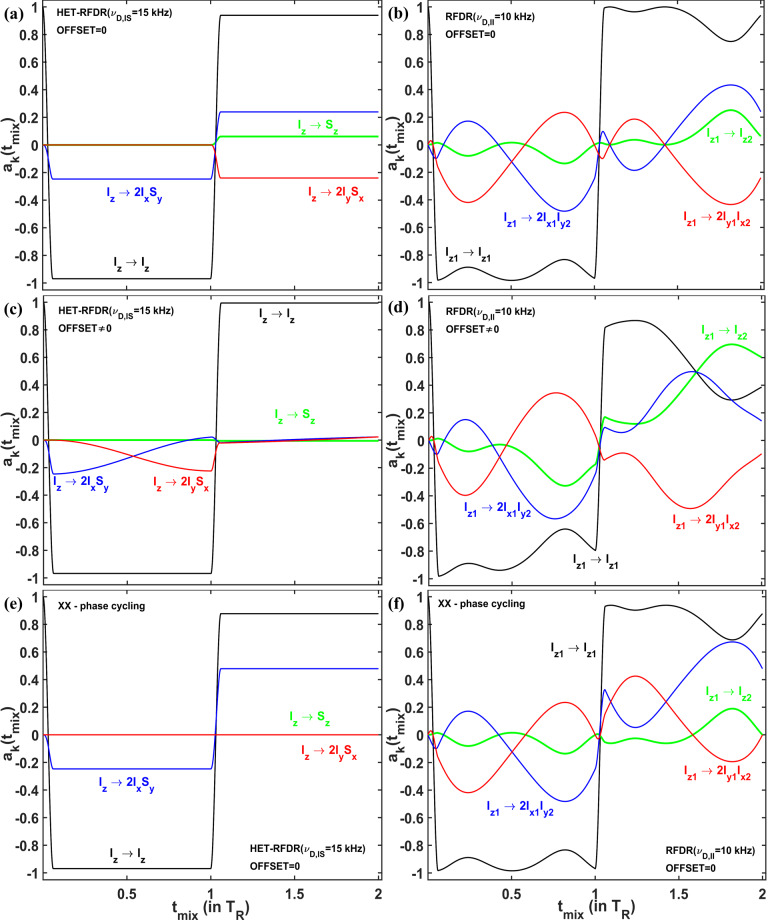
The operator evolution through HET-RFDR and RFDR over two rotor periods. The simulated amplitudes of the operators of a single crystal (Euler angles: 184; 141; 349 ${}^{\circ }$ ) for HET-RFDR **(a, c)** and RFDR **(b, d)**. For the heteronuclear *IS* spin system, $\nu_{D,IS}=15$ kHz, and the initial operator is $I_{z}$ , and for the homonuclear $I_{2}$ spin system, $\nu_{D,\mathrm{II}}=10$ kHz, and the initial operator is $I_{z1}$ . The MAS frequency was 10 kHz, and the rf field was 83 kHz. Black lines – $I_{z}$ and $I_{z1}$ ; green lines – $S_{z}$ and $I_{z2}$ ; blue lines – $2I_{x}S_{y}$ and $2I_{x1}I_{y2}$ ; red lines – $2I_{y}S_{x}$ and $2I_{y1}I_{x2}$ . For **(a–d)**, the phases of the first and second π pulses are X and Y, respectively. Panels **(e, f)** show the case of *IS* and $I_{2}$ spin systems, respectively, when the phases of the first and second π pulses are both X. **(a, b, e, f)** Offset values in kHz: 0 , 0 . **(c, d)** Offset values in kHz: 2 , -3 .

In order to understand via which operators the polarization transfer occurs, we considered the evolution of two systems – $I_{2}$ homonuclear and *IS* heteronuclear spin systems – under RFDR and HET-RFDR sequences with 10 kHz MAS. We simulated the polarization transfers between different operators during the first two rotor periods, which completes the basic RFDR element: $t(\pi_{x})\to {\text{del}}_{1}\to t(\pi_{y})\to {\text{del}}_{2}$ . We consider the amplitudes of the operators for a single molecular orientation since this allows us to see the significant evolution of the operators during the two rotor periods. Figure 5a, c, and e show the amplitudes of four Cartesian operators (Ernst et al., 1987) for *IS* (HET-RFDR), and Fig. 5b, d, and f show the operators for $I_{2}$ (RFDR) spin systems. The measured Cartesian operators are $I_{z}$ , $S_{z}$ , $2I_{x}S_{y}$ , and $2I_{y}S_{x}$ and $I_{z1}$ , $I_{z2},2I_{x1}I_{y2}$ , and $2I_{y1}I_{x2}$ for *IS* and $I_{2}$ spin systems, respectively.

The evolution of four operators *during* two rotor periods for the *IS* spin system and the $I_{2}$ spin system is different, regardless of the offset difference. However, with zero offset difference, the simulated heteronuclear operators (Fig. 5a) and the homonuclear operators (Fig. 5b) show the same values of the amplitudes at one and two rotor periods. From the 64 possibilities (details in the Supplement, in the Operator paths section) for magnetization transfer between heteronuclear operators $I_{z}$ and $S_{z}$ during the two rotor periods, we find only one path with nonzero amplitude: $I_{z}\overset{\pi_{x}}{⟶}2I_{x}S_{y}\overset{{\text{del}}_{1}}{⟶}2I_{x}S_{y}\overset{\pi_{y}}{⟶}S_{z}\overset{{\text{del}}_{2}}{⟶}S_{z}$ . In contrast to the single path found for HET-RFDR, for the homonuclear case, all 64 paths connecting operators $I_{z1}$ and $I_{z2}$ have nonzero amplitudes. However, after each rotor period, the sum of all homonuclear paths provides the same values of the amplitudes as for the heteronuclear *IS* spin system.

In contrast, with a nonzero offset difference, the amplitudes of homonuclear and heteronuclear operators do not coincide at any time (Fig. 5c and d). Moreover, while the amplitude of $I_{z1}\to I_{z2}$ polarization transfer is significantly increased (Fig. 5d, green line), the corresponding heteronuclear amplitude for $I_{z}\to S_{z}$ transfer is significantly decreased (Fig. 5c, green line).

Figure 5c demonstrates the case when negligible small HET-RFDR transfer is observed with $0.5\nu_{R}$ offset difference. To understand the influence of the $0.5\nu_{R}$ offset difference for that case, the evolution of the operators during the first two rotor periods is considered. During the first $\pi_{x}$ pulse, the starting signal is transferred from $I_{z}$ to 2 $I_{x}S_{y}$ . Because of the offset difference of $0.5\nu_{R}$ , the amplitude of this operator is mainly transferred to $2I_{y}S_{x}$ during the first delay (Fig. 5c, red line). Since the second π pulse has phase y , there is no transfer from $2I_{y}S_{x}$ to $I_{z2}$ and very little $I_{z}\to S_{z}$ polarization transfer overall by the end of the second rotor period (Fig. 5c, green line).

In general, for $\pm ∼0.5n\nu_{R}$ ( n=1,3,5,… ) HET-RFDR transfer signal can obtain local minima (negative signals, Fig. S5 in the Supplement), whereas for $\pm ∼n\nu_{R}$ offset differences, local maxima are detected.

The case demonstrated in Fig. 5c indicates the importance of the phase cycling for RFDR and HET-RFDR sequences. Figure 5d and f show the evolution of the operators when there is no offset, and both π pulses have the same phase cycling – XX . For *IS* spin system (Fig. 5e), only two operators have nonzero amplitudes during the investigated time: $I_{z}$ (black line) and $2I_{x}S_{y}$ (blue line), whereas $S_{z}$ and $2I_{y}S_{x}$ are not created. For the $I_{2}$ spin system (Fig. 5d), all four operators evolve during these two rotor periods. However, by the end of two rotor periods, only two operators have nonzero amplitudes, as for the *IS* spin system. In neither case is there magnetization transfer from $I_{z}$ to $S_{z}$ , nor from $I_{z1}$ to $I_{z2}$ after one or two rotor periods. The formal proof of zero transfer signal for the homonuclear two-spin system in the absence of offset differences can be found in the Supplement in the RFDR phase cycling section.

Additional spectra and simulation results are found in the Supplement. We recorded proton–carbon HET-RFDR spectra using fully protonated [ ${}^{13}C$ , ${}^{15}N$ ]-labeled SH3. We numerically simulated multi-spin systems, either containing two protons and two carbons or one nitrogen and two protons, in order to track more complex transfer of magnetization. The main conclusions from the simulations and the experiments in the Supplement are the agreement between experimental and simulated HET-RFDR transfer efficiencies and the expected small dependence of the HET-RFDR recoupling on the flip angle deviations with XY8 phase cycling (Gullion et al., 1990).

## 4 Experimental methods

*Sample preparation.* Microcrystalline chicken alpha-spectrin SH3 protein was used for acquisition of all experimental data. The samples were labeled with 100 % protonation at exchangeable sites and either with alpha proton exchange by transamination ( α -PET) or with uniform ${}^{13}C$ and ${}^{15}N$ labeling with the protocol described in Movellan et al. (2019).

*Simulations.* HET-RFDR and RFDR simulations were performed with in-house MATLAB scripts using the numerical solution of the equation of motion (Nimerovsky and Goldbourt, 2012).

*Solid-state NMR spectroscopy.* The HC and (H)N(H)H spectra of α -PET SH3 were acquired at 14.1 T (600 MHz) using a Bruker AVIIIHD spectrometer using a MASDVT600W2 BL1.3 HXY probe. The experiments were performed at 10 and 55.555 kHz MAS, with the temperature of the cooling gas set to 280 and 235 K, respectively.

For 1D and 2D α -PET SH3 (H)N(H)H spectra, the ramped CP transfer from proton to nitrogen was performed under the same conditions for all experiments: 42.95 kHz on the nitrogen channel and the optimal ramped amplitude on the proton channel of 86.95–108.69 kHz. The mixing time was 1.05 ms. 9.3 kHz WALTZ-16 (Shaka et al., 1983) with 25 µs pulses and 10.4 kHz WALTZ-16 (Shaka et al., 1983) with 100 µs pulses were applied on nitrogen and carbon channels during the acquisition. MISSISSIPPI water suppression (Zhou and Rienstra, 2008) was applied for 100 ms with 13.513 kHz of the rf field. The carrier positions were set to 4.6 ppm, 118.5 ppm, and 53.7 ppm for ${}^{1}H$ , ${}^{15}N$ , and ${}^{13}C$ , respectively, except where otherwise indicated.

Table 1 summarizes the applied experimental parameters for 1D spectra.

**Table 1:** Summary of the experimental parameters used in the 1D CP + RFDR (the start and the end values are shown) and HET-RFDR using α -PET-labeled SH3.

	CP + RFDR		HET-RFDR
	CP	RFDR	
${}^{1}$ H (kHz)	86.95–108.69	86.21	86.21
${}^{15}$ N (kHz)	42.95	–	75.75
Transfer time (ms)	0.55	[0–7.776]	[0–7.776]
NS	32		32
D1 (s)	2		2
AQ (s)	0.020448		0.020448
SW (kHz)	25		25

NS – number of scans; D1 – a recycle delay; AQ – the acquisition time; SW – the spectral width.

For 2D (H)N(H)H HET-RFDR spectra, during the indirect dimension 11.6 kHz, SW ${}_{f}$ –TPPM (Thakur et al., 2006) decoupling with 36.36 µs pulses was applied on the proton channel. Two mixing times were used: 1.152 and 3.456 ms. The widths of π pulses on proton and nitrogen channels were 5.8 and 6.6 µs , respectively. A total of 16 scans were acquired per increment in $t_{1}$ . The total time for the single 2D experiment was 10 h. Table 2 summarizes the rest of the parameters.

**Table 2:** Summary of the experimental parameters used in 2D HET-RFDR α -PET SH3 experiments.

	AQ1; AQ2	SW1; SW2	DW1; DW2
	(s)	(kHz)	( µs )
HET-RFDR	0.0527075; 0.020448	9.713; 25	102.94; 20

1 and 2 are indirect and direct dimensions; AQ – the acquisition time; SW – the spectral width; DW – the dwelling time.

The 2D CP + RFDR experiment with 1.152 and 3.456 ms of mixing time (only 1D slices are shown in Fig. 2b) was performed with the same experimental conditions as 2D HET-RFDR. The CP mixing times from H to N and from N to H were 1.05 and 0.55 ms, respectively.

For all 1D HC HET-RFDR experiments (Fig. 3), 4.8 ms of mixing time was applied. The widths of π pulses on proton and carbon channels were 5.8 µs (86.21 kHz) and 6.6 µs (75.75 kHz), respectively. During the acquisition, 87 kHz SPINAL64 decoupling (Fung et al., 2000) with 6 µs pulses was used. A total of 128 scans were accumulated. The spectral width was 50 kHz and the acquisition time 0.01536 s.

## 5 Conclusion

In this article we firstly demonstrated HETeronuclear RFDR recoupling, when π pulses with XY8 phase cycling were applied simultaneously on two channels. Simultaneous heteronuclear and homonuclear polarization transfers as well as long range contacts were observed in 2D (H)NH spectra using HET-RFDR for the microcrystalline protein SH3 using α -PET labeling. The comparison of 1D HET-RFDR with CP followed by homonuclear RFDR showed similar efficiency of both methods at long mixing times of about 3ms and longer. We experimentally and numerically demonstrated the dependence of the HET-RFDR efficiency on the offset difference between dipolar coupled spins. A numerical operator analysis of both HET-RFDR and RFDR sequences showed that when the offset difference was small with respect to the MAS frequency, and with measurement at a whole number of rotor periods, the behavior of HET-RFDR was similar to the well-known homonuclear RFDR. However, different behaviors were observed when the offset difference could not be neglected.

Considering the evolution of a single crystal during HET-RFDR and RFDR, we showed the operators that were responsible for the transfer. We demonstrated that XY phase cycling of π pulses has a crucial role for both HET-RFDR and RFDR transfer. With phase cycling of XX (or X $\bar{\text{X}}$ ), the transfers between heteronuclear and homonuclear spins did not occur in the absence of offsets. With the presence of the offset differences that cannot be neglected in comparison to the MAS rate, RFDR polarization transfer with phase cycling of XX or X $\bar{\text{X}}$ does occur, although with lower efficiency as was described before (Bennett et al., 1992).

## Supplement

10.5194/mr-2-343-2021-supplement The supplement related to this article is available online at: https://doi.org/10.5194/mr-2-343-2021-supplement.

## Data Availability

The reported NMR data are available upon request.

## Code availability

The simulated MATLAB/Bruker TopSpin codes are available upon request.
