# Supplementary material for: Heteronuclear and homonuclear radio-frequency-driven recoupling
Source: Magn Reson (Gott). 2021 May 28;2(1):343–53. doi: 10.5194/mr-2-343-2021 (PMC10539778; doi:10.5194/mr-2-343-2021)
Supplement: The supplement related to this article is available online at: https://doi.org/10.5194/mr-2-343-2021-supplement. [file mr-2-343-supplement.pdf]

Supplement of Magn. Reson., 2, 343–353, 2021
https://doi.org/10.5194/mr-2-343-2021-supplement
© Author(s) 2021. CC BY 4.0 License.

Open Access
MAGNETIC RESONANCE

Supplement of

Heteronuclear and homonuclear radio-frequency-driven recoupling

Evgeny Nimerovsky et al.

Correspondence to: Loren B. Andreas (land@nmr.mpibpc.mpg.de) and Evgeny Nimerovsky (evni@nmr.mpibpc.mpg.de)

The copyright of individual parts of the supplement might differ from the article licence.

The Supplement consists of four sections. In the first section, “1D HET-RFDR Experiments” we show additional 1D ^{1}H-^{13}C HET-RFDR spectra. The second “HET-RFDR Simulations” section provides additional HET-RFDR simulations, which were performed under conditions that closely match the experiments. The third section, “Operator Paths” shows the possible paths of RFDR and HET-RFDR transfers via heteronuclear and homonuclear operators during the first two rotor periods. The last part, “RFDR Phase Cyling”, shows the formal proof of zero signal transfer for a homonuclear I_2 spin system with zero offset difference and when all π-pulses have the same phase.

1D HET-RFDR Experiments

Figure S1 shows a 1D HET-RFDR pulse sequence. The sequence consists of two π/2-pulses on the ^{1}H channel (with two step phase cycling to eliminate the signal from directly excited spins of carbons) followed by a series of HET-RFDR pulses and finally a π/2-pulse and detection on the ^{13}C channel. The evolution of the magnetization from proton to carbon spins through the HET-RFDR pulse sequence (Figure S1) can be described with cartesian operators as follows:

$$H_z \xRightarrow{first\ proton\ \left(\frac{\pi}{2}\right)_x\ pulse} -H_y \xRightarrow{second\ proton\ \left(\frac{\pi}{2}\right)_{\mp x}\ pulse} \pm H_z$$

$$\xRightarrow{HET-RFDR\ block} \pm a_{HC}(t_{mix})C_z \xRightarrow{first\ carbon\ \left(\frac{\pi}{2}\right)_x\ pulse} \mp a_{HC}(t_{mix})C_y \xRightarrow{detection_{\pm x}} -a_{HC}(t_{mix}), \quad \text{Eq. (S1)}$$

where, $a_{HC}(t_{mix})$, is an amplitude of the transferred signal.

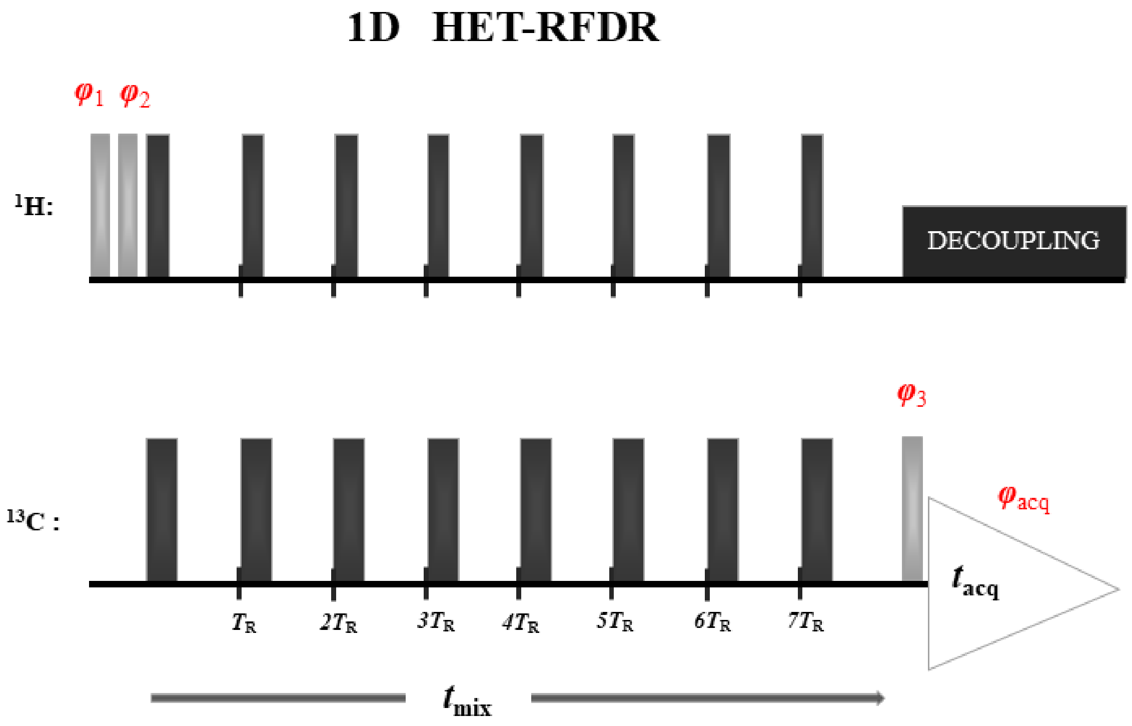

Figure S1 1D HET-RFDR pulse sequence. The sequence consists of two π/2-pulses on the ^{1}H channel, HET-RFDR blocks (a train of π-pulses with a single pulse during each rotor period applied on both channels), π/2-pulse on the ^{13}C channel and detection with proton decoupling. The phases of the π/2-pulses are $\varphi_1 = x$; $\varphi_2 = -x, x$; $\varphi_3 = -x, -x, x, x, y, y, -y, -y$. $\varphi_{acq} = x, -x, -x,\ x, -y, y, y, -y$. π-pulses on the both channels follow the XY8 scheme (Gullion et al., 1990). During acquisition, SW_f-TPPM (Thakur et al., 2006) at 55.555 kHz or SPINAL64 (Fung et al., 2000) at 10 kHz decoupling is applied on the proton channel to narrow the detected resonances.

Figures. S2-S3 demonstrate 1D HC HET-RFDR spectra using [^{13}C, ^{15}N] labeled SH3. On proton and carbon channels π-pulses with different lengths were applied: 3.4 us (147 kHz) and 5 us (100 kHz), respectively. Figure S2a shows HC spectra under different mixing times: 0.576 ms, 1.728 ms, 2.888 ms, 4.032 ms, 5.184 ms and 6.336 ms. Figure S2b shows HC spectra, which were obtained with 6.336 ms HET-RFDR (blue) and RFDR (cyan, π-pulses were applied on carbon channel only) . As expected, HET-RFDR provides ^{1}H to ^{13}C transfer. The efficiency depends on the spectral region. For some aromatic carbons, the transfer\ achieves ~100% efficiency with respect to CP at 1.5 ms (Figure S2b, red), but for other regions, like Cα, the polarization transfer is ~50%.

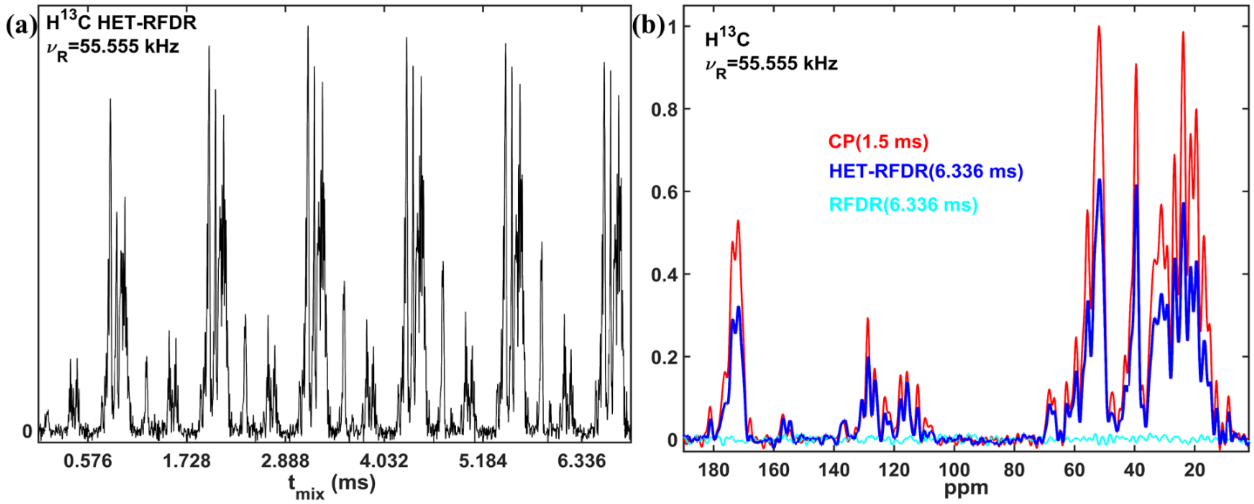

Figure S2 1D HC [^{13}C,^{15}N] labeled SH3 spectra at 55.555 kHz (a) HET-RFDR spectra with different mixing times: 0.576 ms, 1.728 ms, 2.888 ms, 4.032 ms, 5.184 ms, 6.336 ms. (b) Comparaison of 1D HC CP spectrum (red, 1.5 ms of CP mixing) and HET-RFDR spectrum (blue, 6.336 ms of HET-RFDR mixing). The cyan spectrum shows a HC RFDR spectrum, for which π-pulses were applied only on the ^{13}C channel. The carbon refference frequency was set up on 40 ppm. The MAS rate was 55.555 kHz. The experimental parameters are shown in Table S1.

The transfer of the magnetization from H_z operators to C_z operators is minimally affected by flip angle deviations, since XY8 phase cycling is used (Gullion et al., 1990). To show this, we recorded additional 1D HC HET-RFDR spectra with a series of flip angles on the carbon channel. (Figure S3).

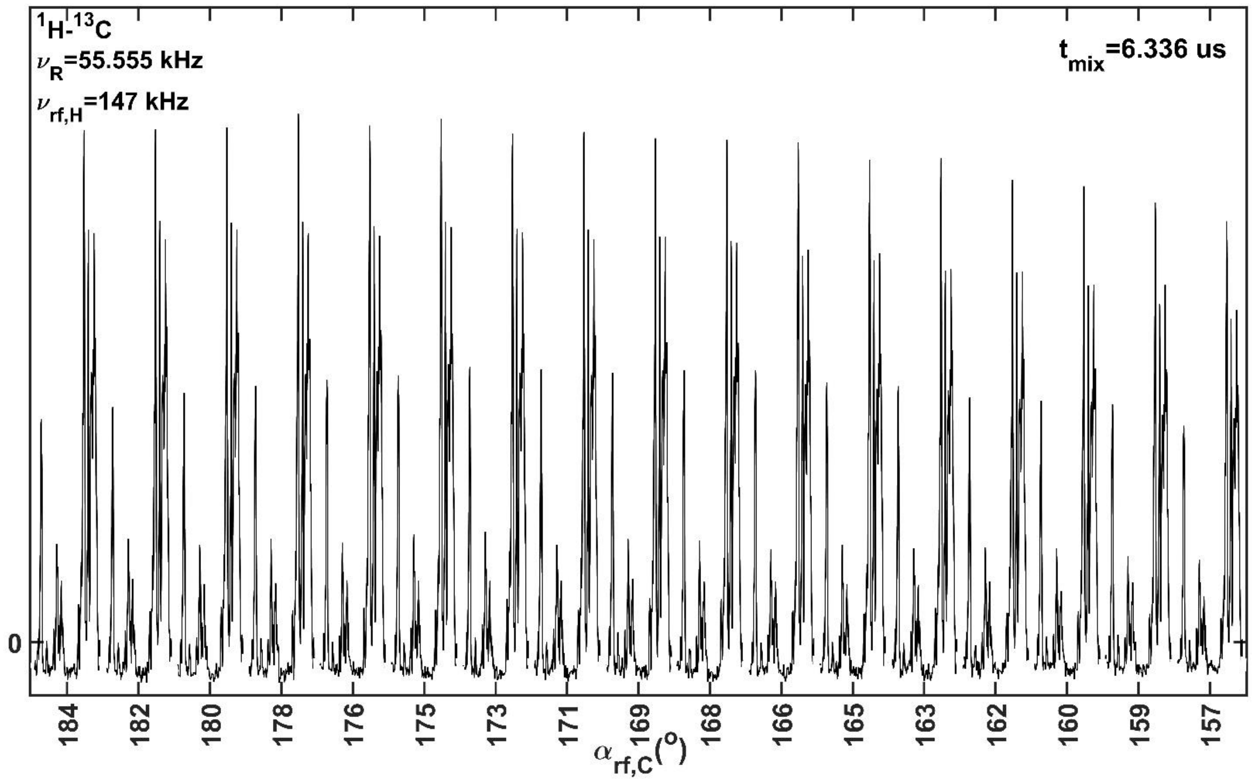

Figure **S3** 1D proton-carbon HET-RFDR spectra of [^{13}C, ^{15}N] labeled SH3 with a 6.336 ms transfer time as a function of the flip angle of the pulses on the carbon channel between 157.34° and 184.64° (17 spectra). The width of π-pulses on the proton channel was 3.4 us. The width of the applied pulses on the carbon channel was constant and equal to 5 us. 55.555 kHz MAS was used. The rf-field values in kHz on the carbon channel from left to right were:87.41, 88.2, 89.01, 89.83, 90.66, 91.51, 92.38, 93.26, 94.16, 95.08, 96.02, 96.97, 97.94, 98.94, 99.95, 100.98, 102.04.

Solid state NMR spectroscopy: The CP and HET-RFDR spectra of ^{13}C,^{15}N SH3 were acquired at 14.1 T (600 MHz) using a Bruker AVIIIHD spectrometer using a MASDVT600W2 BL1.3 HXY probe. The experiments were performed at 55.555 kHz MAS with the temperature of the cooling gas set to 235 K.

For 1D $^1H^{13}C$ spectra during the HET-RFDR periods, the widths of pulses on proton and carbon channels were 3.4 us and 5 us, respectively. 13.89 kHz SW_f-TPPM (Thakur et al., 2006) with 36 us pulses was used during the acquisition. Table S1 summarizes the applied experimental parameters.

Table S1 Summary of the experimental parameters used in the CP (the start and the end values are shown) and HET-RFDR $H^{13}C$ [13C,15N] SH3 experiments.

	CP	HET-RFDR
^{1}H (kHz)	92-115	147
^{13}C (kHz)	43	100, [87.41-102.04]
transfer time (ms)	1.5	[0.576-6.336]
NS	48	48
D1 (s)	1.5	1.5
AQ (s)	0.01536	0.01536
SW (kHz)	50	50

NS – number of scans; D1 – a recycle delay; AQ – the acquisition time; SW – the spectral width.

HET-RFDR Simulations

Figure S4 shows simulated HET-RFDR polarization transfers for four (S_2I_2, Figure S4a) and three (SI_2, Figure S4b) spin systems with conditions that closely match the experiments: 55.555 kHz MAS and 5.4 us π-pulses.

Figure S4a shows the HET-RFDR polarization trasfers between a directly bonded spin pair (I_1-C_2, solid lines) and the remote pair (I_1-C_3, lines with circles). The lines with diamonds represent signals that are not transferred, but remain on the spin I_1. We consider three cases: rigid C_2H_2 chain (black lines), dynamic C_2H_2 chain (red lines) and rigid N_2C_2 chain (blue lines). For the rigid (black solid line) and dynamic (red solid line) C_2H_2 chains when the heteronuclear dipolar coupling constants are larger than the homonuclear dipolar constants, the polarization transfer from H_1 to C_2 oscillates about ~35% efficiency. However, for the spin system with the weak heteronuclear dipolar coupling constants (blue lines), the HET fp-RFDR polarization transfer between directly bonded spins is lower (blue solid line) and achieves only ~20% transfer efficiency.

The signal that remains on the starting spin (lines with diamonds) are ~40% for first two cases (black and solid lines with diamonds) and ~70% for weak dipolar coupling constants (blue line with diamonds).

The HET-RFDR transfer between remote spins, e.g. H_1 and C_3 are about ~10% of the initial polarization for all these three cases (black, red and blue lines with circles). The transfer of magnetization mostly occurs via relayed transfer (I_1-C_2-C_3) and not directly from I_1 to C_3, which more clearly can be seen in the simulations on Figure S4b

Figure S4b considers the HET-RFDR polarization transfer between directly bonded spins (N_1-H_2, solid lines) and remote pair (N_1-H_3, lines with cirles). The heteronuclear dipolar coupling constant between N_1 to H_2 spins is kept constant and the homonuclear dipolar constant between H_2 and H_3 is changed. When the distance between H_2 and H_3 spins is 6 Å (green lines), the fp-RFDR polarization transfer between N_1 and H_2 achieves the maximal transfer of about 50% (solid green line). The direct HET-RFDR polarization transfer efficiency between N_1 and H_3 is very low (green line with circles). For the H_2 - H_3 distances of 4 Å (blue line with circles), 3 Å (red line with circles) and 2 Å (black line with circles), the polarization transfer between N_1 and H_3 achieves ~10%. Since the distance between N_1 and H_3 is not changed, the transfer between N_1 and H_3 is achieved via sequential relayed transfer, N_1-H_2-H_3. The homonuclear distance also has influence on the HET-RFDR polarization tranfer. With decreased H_2-H_3 distance the amplitude of the HET-RFDR polarization tranfer between dirctly bonded spins (solid lines) is decreased.

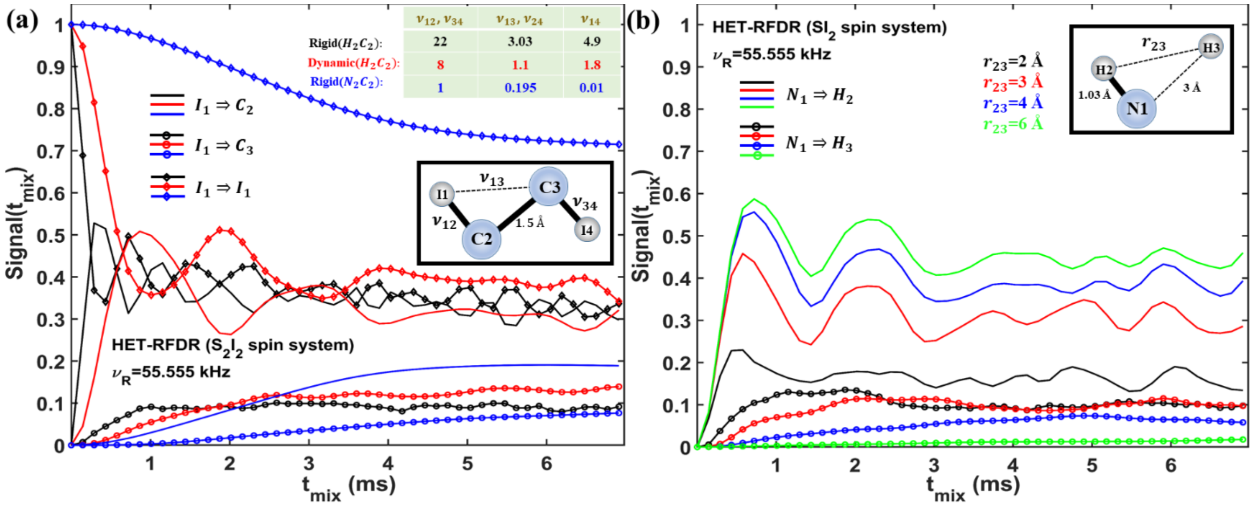

Figure S4 Simulated HET-RFDR signals. The simulated HET-RFDR polarization transfers for S_2I_2 (a) and SI_2 (b) spin systems are shown as a function of mixing time. For all simulations MAS was 55.555 kHz and hard π-pulses with 5.4 us width (92.59 kHz rf-field) were applied simuntaneously every rotor period. The offset and CSA values (the offset and CSA values are defined in the same way as in (Bak et al., 2000)) of spins [I_1;C_2;C_3;I_4] are [1;2;5.5;6] (kHz) and [4;1;2;3] (kHz), respectively. The initial and the final operators were in the direction $\hat{z}$. (a) The solid lines represent the HET-RFDR polarization transfers between I_1 and C_2 spins; the lines with circles represent the HET-RFDR polarization transfers between I_1 and C_3 spins and the lines with diamonds represent the decay of starting signals. The carbon-carbon distance as well as the dipolar coupling constant between C_2

and C_3 were kept unchanged at 1.5 Å (2.22 kHz). The black lines represent the rigid H_2C_2 spin system (with I_1, I_4 of the inset as protons). The proton-carbon dipolar coupling constants were: $\nu_{12} = \nu_{34} = 22\ kHz$; $\nu_{13} = \nu_{34} = 3.03\ kHz$ and the proton-proton coupling constant was: $\nu_{14} = 4.9\ kHz$. The red lines represent the dynamic H_2C_2 spin system with reduced proton-carbon couplings of are: $\nu_{12} = \nu_{34} = 8\ kHz$; $\nu_{13} = \nu_{34} = 1.01\ kHz$ and a reduced proton-proton coupling $\nu_{14} = 1.8\ kHz$. The blue lines represent the rigid N_2C_2 spin system (with I_1, I_4 of the inset as nitrogens). The nitrogen-carbon dipolar coupling constants are: $\nu_{12} = \nu_{34} = 1\ kHz$; $\nu_{13} = \nu_{34} = 0.195\ kHz$ and for the nitrogen-nitrogen coupling, $\nu_{14} = 0.01\ kHz$. (b) The solid lines represent the HET-RFDR polarization trasnfer between N_1 and H_2 spins with unchanged dipolar coupling constant of 11 kHz. The lines with circles represent the transfer between N_1 and H_3 spins for different distances (dipolar coupling constants) between H_2 and H_3 spins: black lines – 2 Å (15 kHz), red lines – 3 Å (4.4 kHz), blue lines – 4 Å (1.9 kHz) and green lines – 6 Å (0.5 kHz). The distance as well as the dipolar coupling constant between N_1 and H_3 were kept unchanged at 3 Å and 0.45 kHz. XY8 phase cycling was used.

Figure S5 demonstrates the simulated HET-RFDR transferred signals for three different spin systems (two, three and four spin systems) with similar offset values as in the experiment ($\Omega_H \approx 0\ kHz$ and $\Omega_C \approx 5\ kHz$, Figure 3f in the main text). In all cases the initial operator was I_z and the measured operator was S_{z1}. For the two spin system (*SI*, black line), the HET-RFDR polarization transfer is negligible. However, for three spins (IS_2, red line) and four spins (IS_3, blue line) the transferred polarization reaches a negative value of -0.05. This suggests the involvement of three or more spins in the transfer, but without a more detailed investigation, it is not immediately obvious via which operators the signal is transferred.

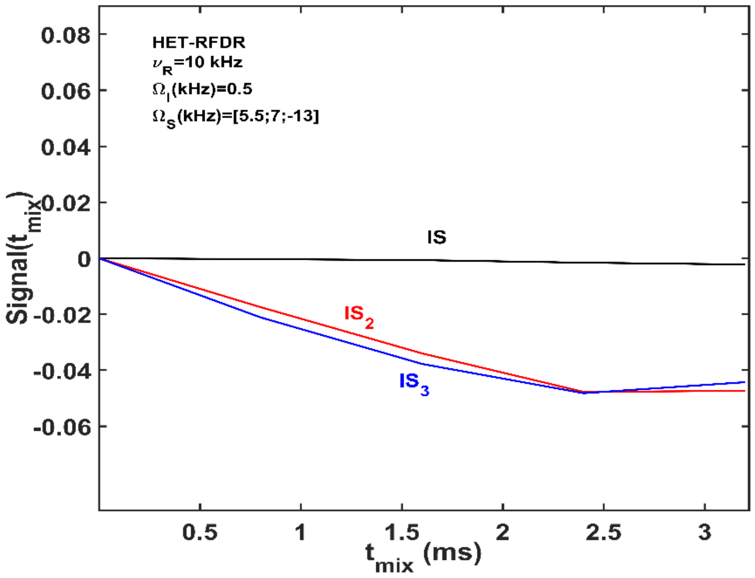

Fig. S5 The simulated HET-RFDR signals. The simulated HET-RFDR polarization transfers for *IS* (black) IS_2 (red) and IS_3 (blue) spin systems as a function of the mixing time. For all simulations, MAS was 10 kHz and hard π-pulses with 5.8 us and 6.6 us widths were applied simuntaneously on *I* and S_n spins every rotor period. The offset and dipolar coupling constants in kHz: *IS* - $[\Omega_I; \Omega_{S1}] = [0.5; 5.5], [\nu_{D,IS1}] = [23]$; IS_2 - $[\Omega_I; \Omega_{S1}; \Omega_{S2}] = [0.5; 5.5; 7], [\nu_{D,IS1}; \nu_{D,IS2}; \nu_{D,S1S2}] = [23; 3.4; 2.5]$; IS_3 – $[\Omega_I; \Omega_{S1}; \Omega_{S2}; \Omega_{S3}] = [0.5; 5.5; 7; -13], [\nu_{D,IS1}; \nu_{D,IS2}; \nu_{D,IS3}; \nu_{D,S1S2}; \nu_{D,S1S3}] = [23; 3.4; 3.2; 2.5; 2.5]$.In all simulations the initial and the measured operators were I_z and S_{z1}, respectively. XY8 phase cycling was used.

Operator Paths

In this section we indentify the paths via which the signals are transferred from I_z to S_z operators and from I_{z1} to I_{z2} operators during the first two rotor periods of HET-RFDR and RFDR blocks, respectively. The simulated parameters were used as in Figure 5a and b. Therefore, these Figures are shown also here as Figure S6.

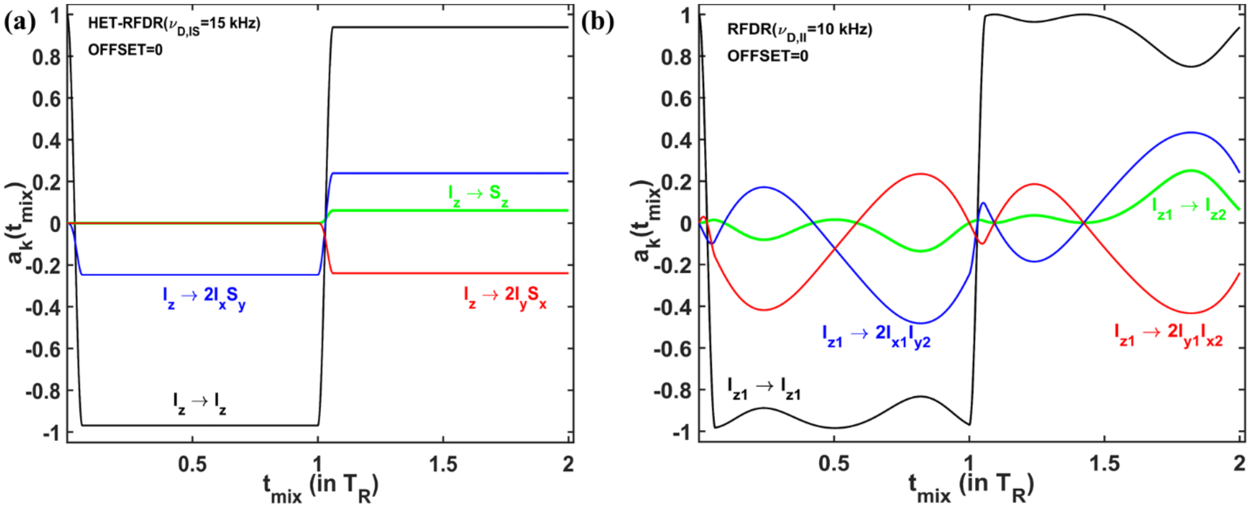

Figure S6 The operator evolution through HET-RFDR and RFDR over two rotor periods. The simulated amplitudes of the operators of a single crystal (Euler angles: 184°; 141°; 349°) for HET-RFDR (a) and RFDR (b). For the heteronuclear *IS* spin system, ($\nu_{D,IS} = 15$ kHz, the initial operator is I_z) and for the homonuclear I_2 spin system, ($\nu_{D,II} = 10$ kHz, the initial operator is I_{z1}). The MAS frequency was 10 kHz and the rf-field was 83 kHz. Black lines – I_z and I_{z1}; Green lines – S_z and I_{z2}; Blue lines – $2I_xS_y$ and $2I_{x1}I_{y2}$; Red lines – $2I_yS_x$ and $2I_{y1}I_{x2}$.

We consider the amplitudes of the operators that are generated as a result of the evolution of the other operators through pulses or dealys: $t(\pi_x) \rightarrow del_1 \rightarrow t(\pi_y) \rightarrow del_2$. We first consider the heteronuclear case of an *IS* spin system during HET-RFDR. Table S2 consists of four subsections. The first, second , third and fourth subsections represent the amplitudes of four operators, I_z, S_z, $2I_xS_y$, $2I_yS_x$, measured at four points.

Table S2 Transfer paths during HET-RFDR. The single crystal amplitudes (Euler angles: 184°; 141°; 349°) of the operators at four time points: π_x – the end of the first pulse; del_1 – the end of the first delay; π_y – the end of the second pulse; del_2 – in the end of the second delay. The first column shows the initial operators. The first, second, third and fourth subsections represent the amplitudes with the initial operators I_z, S_z, $2I_xS_y$, $2I_yS_x$, respectively. The used simulated parameters were as in Figure S6a and in Figure 5a in the main text.

Op	I_z				S_z				$2I_xS_y$				$2I_yS_x$			
	π_x	del_1	π_y	del_2	π_x	del_1	π_y	del_2	π_x	del_1	π_y	del_2	π_x	del_1	π_y	del_2
I_z	**-0.97**	**1**	**-0.97**	**1**	0	0	0	0	0.25	0	0	0	0	0	-0.25	0
S_z	0	0	0	0	-0.97	1	-0.97	1	0	0	-0.25	0	0.25	0	0	0

**2$I_x S_y$**	-0.25	0	0	0	0	0	0.25	0	-0.97	1	-0.97	1	0	0	0	0
**2$I_y S_x$**	0	0	0.25	0	-0.25	0	0	0	0	0	0	0	-0.97	1	-0.97	1

For example, the path $I_z \xrightarrow{\pi_x} I_z \xrightarrow{del_1} I_z \xrightarrow{\pi_y} I_z \xrightarrow{del_2} I_z$ gives the amplitude of $-0.97 \cdot 1 \cdot (-0.97) \cdot 1 = 0.94$ (the bold font in the Table S2), which equals the amplitude of the I_z operator at the end of $2T_R$ in Figure S5a in the main text (black line). The path $I_z \xrightarrow{\pi_x} I_z \xrightarrow{del_1} I_z \xrightarrow{\pi_y} I_z \xrightarrow{del_2} S_z$ gives the amplitude of $-0.97 \cdot 1 \cdot (-0.97) \cdot 0 = 0$. If we analyze all 64 possibilities, we find only one heteronuclear path, conecting I_z and S_z operators through the first two rotor periods of HET-RFDR: $I_z \xrightarrow{\pi_x} 2I_xS_y \xrightarrow{del_1} 2I_xS_y \xrightarrow{\pi_y} S_z \xrightarrow{del_2} S_z$ with nonzero amplitude of $-0.2472 \cdot 1 \cdot (-0.2472) \cdot 1 = 0.061$.

In the same way we tabulate the homonuclear I_2 spin system during the first two rotor periods of RFDR block in Table S3.

Table S3 Transfer paths during RFDR. The single crystal amplitudes (Euler angles: 184°; 141°; 349°) of the operators atfour time points: π_x – the end of the first pulse; del_1 – the end of the first delay; π_y – the end of the second pulse; del_2 – the end of the second delay. The first column shows the initial operators. The first, second, third and fourth subsections represent the amplitudes with the initial operators I_{z1}, I_{z2}, $2I_{x1}I_{y2}$, $2I_{y1}I_{x2}$, respectively. The simulated parameters were as in in Figure S6b and Figure 5b in the main text.

Op	I_{z1}				I_{z2}				$2I_{x1}I_{y2}$				$2I_{y1}I_{x2}$			
	π_x	del_1	π_y	del_2	π_x	del_1	π_y	del_2	π_x	del_1	π_y	del_2	π_x	del_1	π_y	del_2
**I_{z1}**	-0.98	0.97	-0.98	0.97	0.01	0.03	0.01	0.03	0.08	-0.16	-0.16	-0.16	0.16	0.16	-0.08	0.16
**I_{z2}**	0.01	0.03	0.01	0.03	-0.98	0.97	-0.98	0.97	0.16	0.16	-0.08	0.16	0.08	-0.16	-0.16	-0.16
**2$I_{x1}I_{y2}$**	-0.08	0.16	0.16	0.16	-0.16	-0.16	0.08	-0.16	-0.98	0.97	-0.98	0.97	0.01	0.03	0.01	0.03
**2$I_{y1}I_{x2}$**	-0.16	-0.16	0.08	-0.16	-0.08	0.16	0.16	0.16	0.01	0.03	0.01	0.03	-0.98	0.97	-0.98	0.97

Unlike the *IS* spin system, all 64 paths have nonzero amplitudes via which the signal is transferred from homonuclear operator I_{z1} to operator I_{z2} during the first two rotor periods of RFDR. These 64 paths can be divided into four groups.

The first group contains eight paths with combinations of I_{z1}, I_{z2} operators only. For example, the path $I_{z1} \xrightarrow{\pi_x} I_{z1} \xrightarrow{del_1} I_{z1} \xrightarrow{\pi_y} I_{z1} \xrightarrow{del_2} I_{z2}$ has 0.02564 amplitude, whereas the path $I_{z1} \xrightarrow{\pi_x} I_{z2} \xrightarrow{del_1} I_{z2} \xrightarrow{\pi_y} I_{z2} \xrightarrow{del_2} I_{z2}$ has -0.01283092 amplitude. The total amplitude of this group is 0.02561578.

The second group contains 24 paths where each of the paths contains one of the operators $2I_{x1}I_{y2}$ or $2I_{y1}I_{x2}$. For example, the path $I_{z1} \xrightarrow{\pi_x} I_{z1} \xrightarrow{del_1} I_{z1} \xrightarrow{\pi_y} 2I_{x1}I_{y2} \xrightarrow{del_2} I_{z2}$ has -0.02566144 amplitude, whereas the path $I_{z1} \xrightarrow{\pi_x} I_{z1} \xrightarrow{del_1} 2I_{x1}I_{y2} \xrightarrow{\pi_y} I_{z2} \xrightarrow{del_2} I_{z2}$ has 0.01285409 amplitude. The total amplitude of this group is -0.03786813.

The third group contains 24 paths where each of the paths contains two of the operators $2I_{x1}I_{y2}$ or $2I_{y1}I_{x2}$. For example, the path $I_{z1} \xrightarrow{\pi_x} 2I_{y1}I_{x2} \xrightarrow{del_1} 2I_{y1}I_{x2} \xrightarrow{\pi_y} I_{z2} \xrightarrow{del_2} I_{z2}$ has 0.025925 amplitude, whereas the path $I_{z1} \xrightarrow{\pi_x} 2I_{y1}I_{x2} \xrightarrow{del_1} I_{z2} \xrightarrow{\pi_y} 2I_{y1}I_{x2} \xrightarrow{del_2} I_{z2}$ has -0.00073 amplitude. The total amplitude of this group is 0.086262.

The fourth group contains eight paths where each of the paths contains three instances of the operators $2I_{x1}I_{y2}$, $2I_{y1}I_{x2}$. For example, the path $I_{z1} \xrightarrow{\pi_x} 2I_{y1}I_{x2} \xrightarrow{del_1} 2I_{y1}I_{x2} \xrightarrow{\pi_y} 2I_{y1}I_{x2} \xrightarrow{del_2} I_{z2}$ has -0.02566 amplitude, whereas the path $I_{z1} \xrightarrow{\pi_x} 2I_{x1}I_{y2} \xrightarrow{del_1} 2I_{x1}I_{y2} \xrightarrow{\pi_y} 2I_{x1}I_{y2} \xrightarrow{del_2} I_{z2}$ has 0.012854 amplitude. The total amplitude of this group is -0.01262.

The total amplitude of all four groups at the time point $2T_R$ is 0.061, which is the same as for the heteronuclear *IS* spin system.

RFDR Phase Cycling

In this section we show that under the specific conditions of two spins and no chemical shift offsets, there is zero RFDR transfer between operators I_{z1} and I_{z2} at t_{mix}=nT_R (n=1,2,3,…) when XX phase cycling is used. The measured operator at this time is described with the Eq.:

$$\langle I_{z2}\rangle(T_R) = Tr\{I_{z2}U(T_R)I_{z1}U^{-1}(T_R)\}. \qquad \text{Eq. (S2)}$$

We take into account the dipolar interaction as well as the rf-field during the π-pulse. Then the unitary operator, $U(T_R)$ is written as follow:

$$U(T_R) = U_2U_1 \qquad \text{Eq. (S3)}$$

$$U_1 = \hat{T}exp\left\{\int_0^{t_p} dt\left[\omega_{D,12}(t)(3I_{z1}I_{z2} - \bar{I}_1\bar{I}_2) + \omega_{rf}(I_{x1} + I_{x2})\right]\right\}, \quad \text{Eq. (S3a)}$$

$$U_2 = \hat{T}exp\left\{\int_{t_p}^{T_R} dt\omega_{D,12}(t)(3I_{z1}I_{z2} - \bar{I}_1\bar{I}_2)\right\}. \qquad \text{Eq. (S3b)}$$

where $\hat{T}$ is a Dyson operator and $\omega_{D,12}(t)$ is a periodic dipolar time dependent function(Olejniczak et al., 1984) between spins I_1 and I_2. Firstly, we can simplify Eq. S3 omitting the scalar product, $\bar{I}_1\bar{I}_2$, since it commutes with other parts of the Hamiltonian:

$$[\bar{I}_1\bar{I}_2, I_{z1}I_{z2}] = [\bar{I}_1\bar{I}_2, I_{x1} + I_{x2}] = 0, \qquad \text{Eq. (S4)}$$

and the dipolar function is periodic – $\int_0^{T_R} dt\omega_{D,12}(t)\bar{I}_1\bar{I}_2 = 0$. Eq. S3a-b can be written as follow:

$$U_1 = \hat{T}exp\left\{\int_0^{t_p} dt\left[\omega_{D,12}(t)3I_{z1}I_{z2} + \omega_{rf}(I_{x1} + I_{x2})\right]\right\}, \qquad \text{Eq. (S5a)}$$

$$U_2 = \hat{T}exp\left\{\int_{t_p}^{T_R} dt\omega_{D,12}(t)3I_{z1}I_{z2}\right\}. \qquad \text{Eq. (S5b)}$$

The next step is the rotation of all the operators by 90° around axis -y:

$$I_{z1}, I_{z2}, I_{z1}I_{z2}, (I_{x1} + I_{x2}) \xrightarrow{90_{-y}} -I_{x1}, -I_{x2}, I_{x1}I_{x2}, (I_{z1} + I_{z2}). \qquad \text{Eq. (S6)}$$

Substituting Eq. (S6) into Eqs. S2 and Eq. (S5a-b), the modified Eq. (S2) is:

$$\langle I_{z2}\rangle(T_R) = Tr\{I_{x2}U_2U_1I_{x1}U_1^{-1}U_2^{-1}\}, \qquad \text{Eq. (S7)}$$

whereas the modified Eq. (S5a-b) is:

$$U_1 = \hat{T}exp\left\{\int_0^{t_p} dt\left[\omega_{D,12}(t)3I_{x1}I_{x2} + \omega_{rf}(I_{z1} + I_{z2})\right]\right\} \qquad \text{Eq. (S8a)}$$

$$U_2 = \hat{T}exp\left\{\int_{t_p}^{T_R} dt\omega_{D,12}(t)3I_{x1}I_{x2}\right\}, \qquad \text{Eq. (S8b)}$$

The operators in Eq. (S8a-b) can be rewritten with fictitious spin ½ operator formalism(Vega, 1978):

$$2I_{x1}I_{x2} = I_x^{(2,3)} + I_x^{(1,4)}, \qquad \text{Eq. (S9)}$$
$$(I_{z1} + I_{z2}) = 2I_z^{(1,4)} \quad .$$

Therefore, Eqs. (S8a-b) can be written as follow:

$$U_1 = \hat{T}exp\left\{\int_0^{t_p} dt\left[\omega_{D,12}(t)3\left(I_x^{(1,4)} + I_x^{(2,3)}\right) + \omega_{rf}2I_z^{(1,4)}\right]\right\} \qquad \text{Eq. (S10a)}$$

$$U_2 = \hat{T}exp\left\{\int_{t_p}^{T_R} dt\omega_{D,12}(t)3\left(I_x^{(1,4)} + I_x^{(2,3)}\right)\right\}. \qquad \text{Eq. (S10b)}$$

Since the operator $I_x^{(2,3)}$ commutes with other operators and the dipolar function is periodic –

$\int_0^{T_R} dt\omega_{D,12}(t)I_x^{(2,3)} = 0$ – the Eqs. (S7) and (S10a-b) can be rewritten as:

$$\langle I_{z2}\rangle(T_R) = Tr\left\{I_{x2}U_2^{(1,4)}U_1^{(1,4)}I_{x1}\left(U_2^{(1,4)}U_1^{(1,4)}\right)^{-1}\right\}, \qquad \text{Eq. (S11)}$$

$$U_1^{(1,4)} = \hat{T}exp\left\{\int_0^{t_p} dt\left[\omega_{D,12}(t)3I_x^{(1,4)} + \omega_{rf}2I_z^{(1,4)}\right]\right\}, \qquad \text{Eq. (S12a)}$$

$$U_2^{(1,4)} = \hat{T}exp\left\{\int_{t_p}^{T_R} dt\omega_{D,12}(t)3I_x^{(1,4)}\right\}. \qquad \text{Eq. (S12b)}$$

On the basis of the fictitious spin ½ operator formalism(Vega, 1978), the next properties always hold:

$$2I_{xj}I_x^{(1,4)}2I_{xj} = I_x^{(2,3)}, \qquad \text{Eq. (S13)}$$
$$2I_{xj}I_z^{(1,4)}2I_{xj} = -I_z^{(2,3)}, \qquad j = 1,2.$$

On the basis of these properties Eqs. (S11) and (S12) are:

$$\langle I_{z2}\rangle(T_R) = Tr\left\{I_{x2}I_{x1}\breve{U}_2^{(2,3)}\breve{U}_1^{(2,3)}\left(U_2^{(1,4)}U_1^{(1,4)}\right)^{-1}\right\}, \qquad \text{Eq. (S14)}$$

$$\breve{U}_1^{(2,3)} = \hat{T}exp\left\{\int_0^{t_p} dt\left[\omega_{D,12}(t)3I_x^{(2,3)} - \omega_{rf}2I_z^{(2,3)}\right]\right\}, \qquad \text{Eq. (S15a)}$$

$$\breve{U}_2^{(2,3)} = \hat{T}exp\left\{\int_{t_p}^{T_R} dt\omega_{D,12}(t)3I_x^{(2,3)}\right\}. \qquad \text{Eq. (S15b)}$$

On the basis of Eq. (S9) the product of $I_{x2}I_{x1}$ can be rewritten and therefore Eq. (S14) is:

$$\langle I_{z2}\rangle(T_R) = 0.5Tr\left\{\left(I_x^{(2,3)} + I_x^{(1,4)}\right)\left(\breve{U}_2^{(2,3)}\breve{U}_1^{(2,3)}\right)\left(U_2^{(1,4)}U_1^{(1,4)}\right)^{-1}\right\} =$$

$$=0.5Tr\left\{I_x^{(2,3)}\breve{U}_2^{(2,3)}\breve{U}_1^{(2,3)}\right\} + 0.5Tr\left\{I_x^{(1,4)}\left(U_2^{(1,4)}U_1^{(1,4)}\right)^{-1}\right\}. \qquad \text{Eq. (S16)}$$

The next step is to use the properties of fictitious spin ½ operator formalism (Eq. S13) to arrive at:

$$\langle I_{z2}\rangle(T_R) = 0.5Tr\left\{I_x^{(2,3)}\breve{U}_2^{(2,3)}\breve{U}_1^{(2,3)}\right\} + 0.5Tr\left\{I_x^{(2,3)}\left(\breve{U}_2^{(2,3)}\breve{U}_1^{(2,3)}\right)^{-1}\right\}. \qquad \text{Eq. (S17)}$$

The last step is to use the property:

$$-2I_y^{(2,3)}I_x^{(2,3)}2I_y^{(2,3)} = I_x^{(2,3)}, \qquad \text{Eq. (S18)}$$
$$-2I_y^{(2,3)}I_z^{(2,3)}2I_y^{(2,3)} = I_z^{(2,3)}.$$

Substituting Eq. (S18) into Eq. (S115a-b), then the modified Eq. (S15a-b) into Eq. (S17) and considering that $2I_y^{(2,3)}2I_y^{(2,3)} = 1^{(2,3)}$ and $\left[I_x^{(2,3)}, \breve{U}_2^{(2,3)}\right] = 0$, the transferred signal is:

$$\langle I_{z2}\rangle(T_R) = -0.5Tr\left\{I_x^{(2,3)}\left(\breve{U}_2^{(2,3)}\breve{U}_1^{(2,3)}\right)^{-1}\right\} + 0.5Tr\left\{I_x^{(2,3)}\left(\breve{U}_2^{(2,3)}\breve{U}_1^{(2,3)}\right)^{-1}\right\} = 0. \qquad \text{Eq. (S19)}$$

Since the transferred signal is zero at the mixing time of one rotor period, it is always zero at integer multiples of rotor periods.

Bak, M., Rasmussen, J. T., and Nielsen, N. C.: SIMPSON : A General Simulation Program for Solid-State NMR Spectroscopy, J. Magn. Reson. San Diego Calif 1997, 1–35, https://doi.org/10.1006/jmre.2000.2179, 2000.

Fung, B. M., Khitrin, A. K., and Ermolaev, K.: An Improved Broadband Decoupling Sequence for Liquid Crystals and Solids, J. Magn. Reson., 142, 97–101, https://doi.org/10.1006/jmre.1999.1896, 2000.

Gullion, T., Baker, D. B., and Conradi, M. S.: New, compensated Carr-Purcell sequences, J. Magn. Reson. 1969, 89, 479–484, https://doi.org/10.1016/0022-2364(90)90331-3, 1990.

Olejniczak, E. T., Vega, S., and Griffin, R. G.: Multiple pulse NMR in rotating solids, J. Chem. Phys., 81, 4804–4817, https://doi.org/10.1063/1.447506, 1984.

Thakur, R. S., Kurur, N. D., and Madhu, P. K.: Swept-frequency two-pulse phase modulation for heteronuclear dipolar decoupling in solid-state NMR, Chem. Phys. Lett., 426, 459–463, https://doi.org/10.1016/j.cplett.2006.06.007, 2006.

Vega, S.: Fictitious spin 1/2 operator formalism for multiple quantum NMR, J. Chem. Phys., 68, 5518–5527, https://doi.org/10.1063/1.435679, 1978.
